# SIV_SM_/HIV-2 Vpx proteins promote retroviral escape from a proteasome-dependent restriction pathway present in human dendritic cells

**DOI:** 10.1186/1742-4690-4-2

**Published:** 2007-01-09

**Authors:** Caroline Goujon, Lise Rivière, Loraine Jarrosson-Wuilleme, Jeanine Bernaud, Dominique Rigal, Jean-Luc Darlix, Andrea Cimarelli

**Affiliations:** 1 LaboRetro, INSERM U758, Ecole Normale Supérieure de Lyon, IFR 128 BioSciences Lyon-Gerland, Lyon-Biopole, France; 2 Etablissement Français du Sang, Lyon, France

## Abstract

**Background:**

Vpx is a non-structural protein coded by members of the SIV_SM_/HIV-2 lineage that is believed to have originated by duplication of the common vpr gene present in primate lentiviruses. Vpx is incorporated into virion particles and is thus present during the early steps of viral infection, where it is thought to drive nuclear import of viral nucleoprotein complexes. We have previously shown that Vpx is required for SIV_MAC_-derived lentiviral vectors (LVs) infection of human monocyte-derived dendritic cells (DCs). However, since the requirement for Vpx is specific for DCs and not for other non-dividing cell types, this suggests that Vpx may play a role other than nuclear import.

**Results:**

Here, we show that the function of Vpx in the infection of DCs is conserved exclusively within the SIV_SM_/HIV-2 lineage. At a molecular level, Vpx acts by promoting the accumulation of full length viral DNA. Furthermore, when supplied in target cells prior to infection, Vpx exerts a similar effect following infection of DCs with retroviruses as divergent as primate and feline lentiviruses and gammaretroviruses. Lastly, the effect of Vpx overlaps with that of the proteasome inhibitor MG132 in DCs.

**Conclusion:**

Overall, our results support the notion that Vpx modifies the intracellular milieu of target DCs to facilitate lentiviral infection. The data suggest that this is achieved by promoting viral escape from a proteasome-dependent pathway especially detrimental to viral infection in DCs.

## Background

Vpx is a non-structural protein coded by members of the SIV_SM_/HIV-2 lineage, but absent in HIV-1 and in most SIV lineages [1]. Vpx is important for viral replication in macaques [2], but its functions during the early steps of the viral life cycle remain controversial. A number of studies correlated the loss of nuclear localization of Vpx with the inability of mutant viruses to infect non-dividing cells, thus arguing for its role in nuclear import, similarly to HIV-1 Vpr [3-12]. However, several studies indicated that Vpx localization was more complex and that Vpx-deficient mutants were defective independently of the cells' cycling status, suggesting a function other than nuclear import [13-19]. It is highly possible that these discrepancies result from the heterogeneity of the experimental systems used. None of the previous studies examined the function of Vpx in the infection of DCs.

We have previously shown that in a single round infectivity assay, Vpx is absolutely required for the infection of human monocyte-derived DCs by SIV_MAC _LVs, while it is largely dispensable for the infection of other non-dividing cell types [20,21]. Interestingly, we have also shown that Vpx can be functionally provided *in trans *by pre-incubation of DCs with non-infectious SIV_MAC _VLPs. In this setting, VLPs composed of viral structural and accessory proteins but devoid of viral genome are simply used as carriers of those viral proteins that are normally delivered in target cells upon viral infection. By analyzing VLPs of different composition, we have determined that Vpx is the sole viral protein required for the positive effect of SIV_MAC _VLPs (named hereafter Vpx-VLPs, [20]). Upon pre-incubation, Vpx increased the infectivity of the closely related HIV-1 lentiviral vector by at least 10-fold [20]. This effect was specific for DCs and to a milder extent for macrophages and occurred in the absence of detectable changes in DCs physiology.

Here, we investigated Vpx functions at a molecular level and showed that Vpx proteins derived from different strains of the SIV_SM_/HIV-2 lineage act by promoting the rapid accumulation of full length viral DNA following infection with a wide variety of retroviruses. More importantly, we discovered that MG132, a known proteasome inhibitor, partially rescues the defect of Vpx-deficient SIV_MAC _LVs and displays effects similar and non-additive to Vpx in the infection of DCs by HIV-1.

## Results

### The positive effect of Vpx in lentiviral infection of human DCs is a unique property of members of the SIV_SM_/HIV-2 lineage

**Figure 1:**
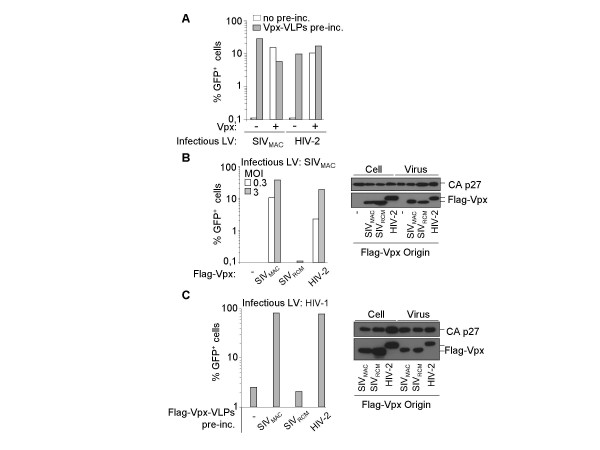
**The function of Vpx in the infection of DCs is conserved uniquely in members of the SIV_SM_/HIV-2 lineage**. A) HIV-2 and SIV_MAC _LVs rely on Vpx for the infection of DCs. VSVg-pseudotyped SIV_MAC _and HIV-2 LVs (coding or not for Vpx) were produced in 293T cells, purified by ultracentrifugation and used to infect DCs at a multiplicity of infection (MOI) of 3. Vpx-containing SIV_MAC _VLPs (Vpx-VLPs) similarly produced were added onto DCs at MOI equivalent of 2 (as measured by exo-RT test with standards of known infectivity) for 2 hrs prior to infection with the above-mentioned LVs. GFP^+ ^cells were scored 3 days later by flow cytometry. B) Only Vpx proteins from the SIV_SM_/HIV-2 lineage rescue the infectivity defect of Vpx-deficient SIV_MAC _LVs. VSVg-pseudotyped SIV_MAC _LVs (SIV15^-^, coding *gag-pro-pol*) were produced in presence or absence of Flag-tagged Vpx proteins derived from SIV_MAC_, SIV_RCM _and HIV-2. Virions were then normalized for their infectious titer on HeLa cells and used to infect DCs at MOI 0.3 or 3. The incorporation of Flag-Vpx proteins into virion particles was assessed by Western blot (right panel). The different migration on SDS-PAGE of HIV-2 and SIV_MAC _Vpx proteins has already been reported [40]. C) Only Vpx proteins from the SIV_SM_/HIV-2 lineage increase WT HIV-1 LVs infection in a pre-incubation assay. Non-infectious SIV_MAC _VLPs containing the different Flag-Vpx proteins were produced and used as described in A in a pre-incubation assay to test their effect on WT HIV-1 LVs infectivity (used at a constant MOI of 3). Incorporation of Flag-Vpx proteins into VLPs was assessed by Western blot (right panel). One representative data set out of 3 to 5 independent experiments is shown for each panel.

To determine if the requirement for Vpx in the infection of DCs was conserved in other members of the SIV_SM_/HIV-2 lineage, DCs were infected with SIV_MAC _and HIV-2 LVs coding or lacking Vpx. Cells were analyzed 3 days later by flow cytometry to score GFP positive infected cells (Fig. 1A). HIV-2 LVs were capable of infecting DCs but relied on the presence of Vpx, as we previously reported for SIV_MAC _LVs [20].

Given that Vpx rescues the infectivity defect of Vpx-deficient SIV_MAC _LVs when supplied in target cells via non-infectious Vpx-VLPs, we sought to determine if pre-incubation could similarly rescue Vpx-deficient HIV-2 LVs (Fig. 1A). Pre-incubation of DCs with Vpx-VLPs had only marginal effects on the efficiency of infection of complete (Vpx-containing) HIV-2 and SIV_MAC_LVs (1.5–2 fold positive and a 1.5–2 fold decrease, respectively). On the contrary, pre-incubation completely rescued the defect of Vpx-deficient HIV-2 LVs, demonstrating that HIV-2 and SIV_MAC _Vpx proteins have conserved functions.

To extend our observation further, the Vpx proteins of SIV_MAC _and HIV-2 were compared with the one derived from the red capped mangabey SIV (SIV_RCM_) with which they share only 30% sequence identity. Proteins were flag-tagged at their N-terminus, as no available antibody allowed SIV_RCM _Vpx detection. The ability of these proteins to functionally replace SIV_MAC _Vpx was first assayed in the context of SIV_MAC _LVs infection (Fig. 1B). Vpx proteins were co-expressed along with minimal SIV_MAC _LVs (SIV15-, coding only *gag-pro-pol*) and virion particles were purified and normalized. DCs were then infected at high and low viral inputs (Fig. 1B, MOIs 0.3 and 3, as assessed on HeLa cells). Despite being well incorporated into virion particles (right panel, as indicated), SIV_RCM _Vpx was unable to functionally complement the infectivity defect of Vpx-deficient SIV_MAC _LVs. Similarly, SIV_RCM _Vpx-containing VLPs had no positive effect on the infectivity of WT HIV-1 LVs in a typical pre-incubation assay (Fig. 1C). As we had previously shown, similar effects were observed with WT or Vpr-deficient HIV-1 vectors, suggesting that Vpr didn't share similar functions than Vpx (not shown and [20]).

Given that Vpx proteins derived from other strains of the SIV_SM_/HIV-2 lineage tested behaved as shown for HIV-2 and SIV_MAC _(not shown), these results suggest that the function of Vpx in the infection of DCs is unique to members of this lineage.

### Vpx allows the accumulation of full length viral DNA following SIV_MAC _LVs infection of DCs

**Figure 2:**
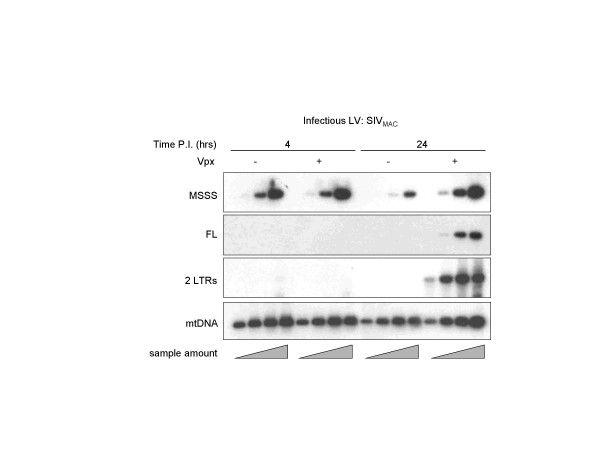
**Vpx allows the accumulation of full length viral DNA following SIV_MAC _infection of DCs**. DCs were infected with normalized amounts of SIV_MAC _LVs containing or not Vpx (MOI of 2). Cell aliquots were harvested at 4 and 24 hrs post-infection and analyzed by semi-quantitative PCR (serial five-fold sample DNA dilutions) using primers that recognized specifically early (MSSS) and late (FL and 2LTRs) products of reverse transcription. The amount of sample added in the PCR reaction decreases from right to left, as represented by triangles: sample amount). Amplification of mitochondrial DNA (mtDNA) was used for normalization. PCR products were transferred onto a nylon membrane and hybridized with ^32^P-labelled specific probes prior to phosphor imager analysis and quantification. One representative data set out of 4 independent experiments is shown here.

To dissect the effects of Vpx at a molecular level, the accumulation of reverse transcription intermediates was analyzed by semi-quantitative PCR on DCs lysates obtained upon infection with SIV_MAC _LVs containing or not Vpx (Fig. 2). PCR products were transferred onto a nylon membrane, hybridized with ^32^P-labelled specific probes and analyzed by phosphor imager quantification.

Early RT products (minus strand strong stop, MSSS) were readily detected, although a minor defect in MSSS accumulation was observed at 24 hrs post infection in the absence of Vpx (2.5 fold after mtDNA normalization). In contrast, accumulation of full length (FL) viral DNAs was drastically reduced in the absence of Vpx (at least 100 fold). Not surprisingly, no episomal 2LTRs forms were detected in this case.

These results strongly suggest that Vpx is required in DCs for the accumulation of full length viral DNA during the early steps of SIV_MAC _infection.

### Vpx has a wide positive effect on viral infection of DCs and acts by promoting the accumulation of full length viral DNA

**Figure 3:**
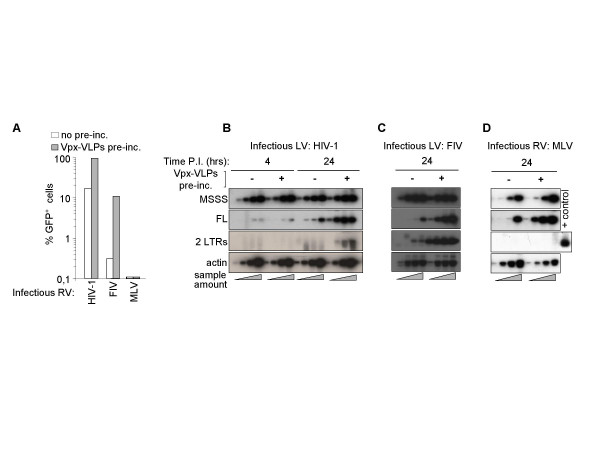
**Vpx exerts a general positive effect on lentiviral infection and results in an increased accumulation of full length viral DNA**. A) Infections of DCs were carried out with VSVg-pseudotyped retroviral vectors bearing a CMV-GFP expression cassette (RVs, MOI 5) with or without Vpx-VLPs pre-incubation (MOI equivalent of 2, measured by exo-RT activity in comparison with standards of known infectivity). The percentage of infected cells was determined by flow cytometry 72 hours afterwards. B) DCs were pre-incubated with Vpx-VLPs at an MOI equivalent of 2 for 2 hrs prior to infection with a constant amount of HIV-1 (B), FIV (C) and MLV retroviral vectors (RV, D) at MOI 5. Cell aliquots were harvested at 4 and 24 hrs post-infection for HIV-1 and at 24 hrs only for FIV and MLV and analyzed by semi-quantitative PCR on serial five-fold sample dilutions (sample amount represented by triangles, as in the legend to Fig. 2), using primers that recognized specifically early and late products of reverse transcription. Amplification of actin DNA (actin) was used for normalization. For MLV, the positive control for 2LTRs amplification is represented by cell lysates of HeLa cells obtained 24 hrs post-infection with 10 fold less MLV vector than was used for DCs. PCR products were transferred onto a nylon membrane and hybridized with ^32^P-labelled specific probes prior to phosphor imager analysis and quantification. One representative data set out of 3 to 4 independent experiments is shown here.

To explain the positive effect of Vpx on the efficiency of infection of an heterologous virus (HIV-1, [20]), two hypotheses were put forward: Vpx could bind to a conserved viral element or it could associate with cellular proteins that modulate viral infection specifically in DCs. To distinguish between these possibilities, the effect of Vpx-VLPs pre-incubation was evaluated on the infectivity of a larger panel of retroviral vectors (HIV-1 as control, the feline immunodeficiency virus, FIV, and the murine leukemia gammaretrovirus, MLV). Cells were exposed to Vpx-VLPs for 2 hours prior to infection with an equal amount of infectious GFP-coding vectors and flow cytometry analysis was carried out 3 days later (Fig. 3A). In the absence of pre-incubation, HIV-1 LVs infected DCs at a much higher rate than FIV LVs, while MLV vectors were totally non-infectious. Vpx-VLPs pre-incubation of DCs strongly increased both HIV-1 and FIV LVs infection efficiencies (from 10 to 95% and from virtually undetectable to 10%, respectively), while MLV remained non-infectious, as previously shown [22].

To characterize the effect of Vpx on heterologous viruses infection, the accumulation of viral DNA products was examined (Fig. 3B, 3C and 3D). Vpx-VLPs pre-incubation dramatically increased the levels of FL viral DNA at 24 hrs following HIV-1, FIV and surprisingly also MLV infection, despite the presence of similar levels of MSSS (Fig. 3B, 3C, 3D, from 10 to 30-fold depending on the virus). For HIV-1 and FIV, the increase in 2LTR DNA was proportional to the increase of FL DNA. In the case of MLV, the observed increase in late RT products didn't result in the ability of the virus to infect DCs. The absence of circular 2LTR forms, indicative of viral DNA passage into the nucleus, suggests that a major nuclear import block exists for MLV in DCs that acts successively or dominantly over Vpx.

Overall, these results indicate that Vpx promotes the accumulation of full length viral DNA in DCs following homologous as well as heterologous retrovirus infection. Given the low sequence conservation between viral elements of MLV, FIV and HIV, we believe these results strongly argue that Vpx modifies the intracellular environment of DCs to the virus advantage.

### Vpx increases the kinetic of infectious viral DNA accumulation in DCs

**Figure 4:**
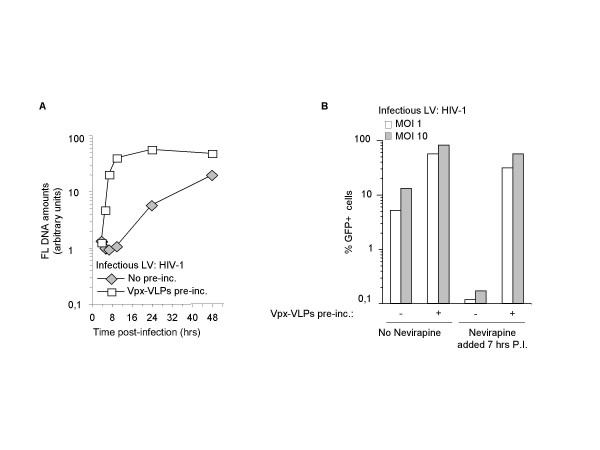
**Vpx allows faster rates of complete and infectious viral DNA accumulation following HIV-1 infection**. A) DCs were infected with a constant amount of HIV-1 LVs with or without Vpx-VLPs pre-incubation. Cell aliquots were harvested at times comprised between 4 and 48 hrs post-infection and the accumulation of FL viral DNA analyzed by semi-quantitative PCR. PCR products were quantified by phosphor imager (ordinate) after southern blot and hybridization analysis and input DNA normalization and are presented here in function of time (abscissa). B) DCs were infected with HIV-1 LVs with or without Vpx-VLPs pre-incubation and infection's rates obtained under normal conditions were compared with those obtained by inhibiting RT synthesis by addition of the nonnucleoside RT inhibitor Nevirapine at 7 hrs post-infection (10 μg/ml, this concentration inhibits completely viral infection if provided at the time of infection, not shown). GFP positive cells were analyzed by flow cytometry 5 days post infection. One representative data out of 2 independent experiments are presented for each panel.

Viral DNA accumulation most likely relies on multiple factors such as RT synthesis rates and viral nucleoprotein complexes stability or trafficking to favorable intracellular locations. To gain further insights into Vpx function, a more detailed time course analysis of complete reverse transcripts accumulation was carried out on DCs infected with HIV-1 LVs with or without Vpx-VLPs pre-incubation (Fig. 4A). HIV-1 was chosen because Vpx had important effects on its infectivity and because reverse transcription could be blocked with Nevirapine, a potent reverse transcriptase inhibitor (see below). In the absence of pre-incubation, FL DNA accumulation proceeded rather slowly for the first 7 hrs of infection and increased linearly thereafter up to 48 hrs. On the contrary, in presence of Vpx-VLPs pre-incubation HIV-1 FL viral DNA accumulated rapidly within the first 7 hrs and increased only marginally thereafter. By 48 hrs post-infection the overall amounts of FL viral DNA attained similar levels in both conditions (within 2–3-fold as opposed to the 20-fold difference observed at 7 hrs). However, despite the fact that similar levels of FL viral DNA were reached at 48 hrs post-infection, HIV-1 infection rates were much higher upon Vpx-VLPs pre-incubation (see for example Fig. 1C and 3A).

To prove that viral genomes synthesized early in presence of Vpx are truly infectious and that they have an advantage over DNA produced at later times, DCs were infected with HIV-1 LVs (at MOI 1 and 10) in presence or absence of Vpx-VLPs pre-incubation. Infections were blocked after 7 hrs with the nonnucleoside inhibitor Nevirapine and compared to untreated samples 5 days post-infection by flow cytometry (Fig. 4B). Nevirapine is a potent RT inhibitor and blocks efficiently viral infection. However, the drug has no effect on the migration, integration and expression of already completed viral DNA. Our analysis indicates that contrarily to WT, the majority of viral genomes has already been completed by 7 hrs post-infection. Indeed, the percentage of GFP^+ ^cells is similar if the drug is absent or added at this early time point.

### Vpx-mediated accumulation of full length viral DNA occurs independently from arsenate

**Figure 5:**
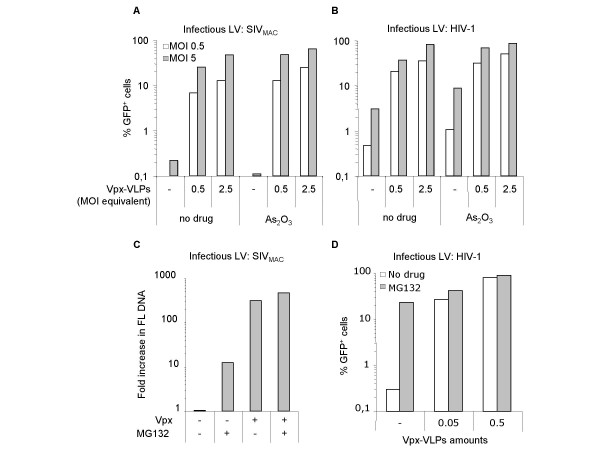
**Vpx and the proteasome inhibitor MG132, but not Arsenate, display similar effects on the infection of DCs**. A) DCs were either infected with Vpx-deficient SIV_MAC _or HIV-1 LVs (A and B, respectively, at MOI of 0.5 and 5) and treated singularly or in combination with 1 μM arsenic acid (As_2_O_3_) and Vpx-VLPs (at MOI equivalents of 0,5 and 2,5). The efficiency of infection was evaluated 72 hrs after by flow cytometry analysis. C) DCs were infected with Vpx containing or deficient SIV_MAC _LVs (MOI 5) in presence or absence of MG132 (1 μg/ml). The drug was added 30 min prior to infection then left for a total of 7 hrs prior to cell washing and media replacement. DCs were then lysed at 24 hrs post-infection for FL PCR analysis as described in the legend of Fig. 5A. Results are presented as a fold increase in FL DNA for each condition with respect to the amount produced upon infection with Vpx-deficient SIV_MAC _LVs. D) DCs were similarly infected with a constant amount of HIV-1 LVs in presence or absence of MG132 and 2 different amounts of Vpx-VLPs. Media was replaced 7 hrs post-drug addition and cells analyzed 2 days afterwards by flow cytometry. One representative experiment out of 3 is shown here for each panel.

Arsenic acid is a drug known to enhance reverse transcription efficiency in certain cell types by an unknown mechanism [23]. As Vpx enhances also viral DNA accumulation, we sought to determine if Vpx and arsenate acted along the same pathway. The possible effects of arsenate and of Vpx-VLPs pre-incubation of DCs were determined on SIV_MAC _LVs lacking Vpx or HIV-1 LVs (Fig. 5A and 5B, respectively). Arsenic acid did not rescue the infectivity defect of SIV_MAC _LVs devoid of Vpx, but increased the efficiency of infection to a mild extent when Vpx was present. Arsenic acid increased as well HIV-1 infectivity independently of Vpx-VLPs pre-incubation. In both cases, the effect of arsenic acid on viral infectivity was negligible (from 1.5 to 3 fold increase) when compared to the effect of Vpx. These results suggest that Vpx promotes viral DNA accumulation via a separate mechanism.

### Vpx counteracts a proteasome-dependent pathway in DCs

Since proteasome inhibitors have been shown to influence viral infectivity by modulating the accumulation and stability of viral DNAs [24-28], we sought to determine if Vpx could interfere with this pathway. The proteasome inhibitors MG132, lactacystine and epoxomicin were initially tested, but only MG132 was retained due to its lower toxicity. Even so, DCs remained viable only if MG132 treatment was limited to a very short time (7 hrs at 1 μg/ml). To determine if MG132 could rescue the infectivity defect of Vpx-deficient SIV_MAC _LVs, DCs were infected in presence or absence of the drug for 7 hrs prior to media replacement and drug and virus removal. If cells were analyzed by flow cytometry 2 days later, MG132 treatment didn't consistently rescue the infectivity defect caused by the absence of Vpx (data not shown). We hypothesized this could be due to the drastic defect of Vpx-deficient SIV_MAC _LVs and to the short time of treatment to which the experiments were constrained. However, if infections were analyzed by PCR 24 hrs post-infection, MG132 partially relieved the block in FL viral DNA accumulation (by 12.5-fold, Fig. 5C). On the contrary, MG132 had only a marginal effect on the amount of viral DNA accumulated in presence of Vpx (1–2 fold). Thus, MG132 induced FL DNA accumulation similarly but not additively to Vpx. It may be possible that a more complete restoration of FL DNA levels could have been obtained for LVs devoid of Vpx with longer exposures or higher concentrations of MG132 and that this could have yielded to a consequent detection of GFP-positive cells by flow cytometry. However, the toxicity of the drug on DCs precluded these possibilities.

Given that the defect of Vpx-deficient SIV_MAC _LVs was rather drastic, we hypothesized that the interplay between MG132 and Vpx could be better revealed in more permissive conditions such as in the context of pre-incubation assays (Fig. 5D). DCs were infected with a constant amount of HIV-1 LVs and treated with MG132 in presence or absence of Vpx-VLPs (at MOI equivalents of 0.05 and 0.5). In the absence of Vpx-VLPs pre-incubation, MG132 increased the infectivity of HIV-1 to levels achieved upon VLPs pre-incubation (30-fold on average). As shown above for SIV_MAC_, MG132 had only marginal effects on HIV-1 infectivity when Vpx-VLPs were present, suggesting that Vpx and MG132 act by similar mechanisms. These results suggest that the function of Vpx in the infection of DCs may be to counteract a proteasome-dependent restriction.

## Discussion

Our data support the notion that Vpx of the SIV_SM_/HIV-2 lineage allows the efficient accumulation of complete viral DNA by counteracting a proteasome-dependent restriction pathway specifically in DCs. We have not observed a similar role of Vpx in the infection of other non-dividing cell types such as macrophages or IL7-stimulated PBLs, although Vpx had a minor stimulating effect on the former cell type [20]. This suggests that the restriction pathway that is targeted by Vpx is particularly active in DCs with respect to other cell types.

We believe that multiple evidences support the hypothesis that Vpx modifies DCs by counteracting a specific restriction mechanism. Vpx provided *in trans *in target DCs induces the accumulation of viral DNAs following infection with quite distantly related retroviruses, excluding the possibility that Vpx acts on conserved viral elements. This function of Vpx is specific to immature DCs (as well as mature DCs, not shown). Lastly, the positive effect of Vpx is maintained if Vpx-VLPs and infectious LVs enter DCs via distinct entry pathways (RD114, GALV and VSVg, not shown), supporting the notion that Vpx targets cellular rather than viral components.

The kinetic analysis of full length viral DNA accumulation following HIV-1 infection revealed that Vpx speeds up the completion of the RT process, a reaction that seems relatively slow in DCs. Indeed, the majority of viral DNA is synthesized by 7 hrs in presence of Vpx as opposed to 48 hrs in its absence. Despite the fact that at 48 hrs post-infection equivalent amounts of viral DNA accumulated in both conditions, the viral DNA synthesized in presence of Vpx is by far more infectious. This suggests that viral genomes that are not completed in a short time are more likely to be targeted by anti-viral cellular defense mechanisms that diminish their infectivity. Given that the viral DNA is contained within a nucleoprotein complex that chaperones it through its life cycle, such defenses may act at multiple steps. In this respect, by promoting the completion of viral DNA synthesis by RT, Vpx may drive structural rearrangements in viral complexes that alter their stability or their trafficking within the cytoplasm with the result of protecting them.

Several results shown here argue that the block relieved by Vpx in DCs utilizes the proteasome. In fact, the proteasome inhibitor MG132 partially rescued the accumulation of full length viral DNA after infection with SIV_MAC _LVs lacking Vpx. MG132 had an effect of the same order of magnitude of Vpx on HIV-1 infection but the two effects were not additive. Lastly, the positive effect of proteasome inhibitors on viral infection of most cell types appears much milder than the one observed here in DCs ([24-28] between 3 to 7 fold, as opposed to 30 fold on average in DCs). Due to their high antigen processing ability, the possibility that DCs display high levels of proteasome activity is not unlikely. Although this hypothesis remains to be tested, it may explain why Vpx is required specifically in DCs.

An alternative explanation for the phenomenon observed here is that Vpx does not target a restriction pathway but simply increases the overall efficiency of RT synthesis by altering the intracellular dNTP pool. Although such hypothesis has not been tested directly, we believe it unlikely because early RT products (MSSS) are unaffected by Vpx.

A Vif-insensitive restriction block specified by APOBEC3G molecules present in the form of low molecular weight complexes has been described in cells resistant to HIV-1 infection, such as quiescent lymphocytes, monocytes and more recently DCs [29,30]. However, Vpx doesn't restore HIV-1 infection in quiescent lymphocytes nor monocytes (not shown) making it unlikely, although formally possible, that Vpx acts by inhibiting APOBEC.

Our data may be reminiscent of the tripartite motif protein 5alpha-induced restriction (TRIM5α) and of its negative impact on lentiviral infection [31,32]. However, we believe that the effect described here are independent from TRIM5α-mediated restriction. Indeed, the defect of Vpx-deficient SIV_MAC _LVs is not relieved with increasing amounts of viral targets and human TRIM5α is not known to target HIV-1 nor SIV_MAC _infection. This suggests that Vpx may act by counteracting a distinct restriction pathway that remains to be identified.

## Conclusion

Vpx is required for viral spread and dissemination of SIV_SM _in macaques. Our data indicates that Vpx exerts a unique function in lentiviral infection of DCs by promoting a rapid accumulation of complete viral DNA forms and in mediating the escape of viral genomes from a proteasome-dependent pathway that restricts viral infection in such cells. Given the central role of DCs in viral spread, these results may partly explain the drastic phenotype of Vpx mutants *in vivo*.

## Methods

### Cells

Human primary lymphocytes and monocytes were obtained from peripheral blood mononuclear cells (PBMCs) of healthy donors at the Etablissement Français du Sang de Lyon [33]. Monocytes obtained by negative selection to more than 95% purity (MACS microbeads, Miltenyi Biotec), were further differentiated in immature dendritic cells (DCs) upon culture for 4–6 days in GM-CSF/IL4 (100 ng/ml [34]). Human 293T were maintained in complete DMEM plus 10% FCS. When indicated, arsenate was used at 1 μM and left on cells throughout the experiment. MG132 (SIGMA) was used at 1 μg/ml for a total of 7 hrs prior to media replacement and was added 30 min prior to infection.

### Retroviral vectors

The HIV-1, SIV_MAC251 _(SIV_MAC _in the text), HIV-2 and FIV-based lentiviral vectors, as well as the murine leukemia virus (MLV) retroviral vector have been described elsewhere [21,35-38]. They share similar conceptions and are obtained upon transfection with: packaging constructs coding *gag-pro-pol *and viral accessory proteins; a miniviral genome bearing a CMV-GFP expression cassette; and a vesicular stomatitis virus G envelope protein (VSVg) that confers them ample cellular tropism [21,22,35,38,39]. Retroviral vectors and non infectious virion-like-particles (lacking therefore a viral genome but otherwise identical to infectious particles) were produced by calcium phosphate DNA transfection of 293T cells and purified by ultracentrifugation through a double-step sucrose cushion (45/25% w/v, as in ref [33]). Virions were normalized by exogenous reverse transcriptase assay (exo-RT) with standards of known infectivity or by determining their infectious titers on HeLa cells and no appreciable differences were observed between the two methods. Infections were carried out for 2 hrs prior to cell washing and cells examined 72 hours after by flow cytometry, unless otherwise specified. Routine control infections were performed with RT inhibitors to exclude pseudotransduction.

The SIV_MAC _Vpx-deficient packaging construct has been described previously (SIV15-, [21]). The HIV-2 Vpx-deficient packaging construct was derived from the initial pSVRΔNB construct [39], by introducing a deletion encompassing nucleotides 69–283 of *vpx *by digestion with the unique enzyme NsiI present within its sequence and Bal31 nuclease digestion (HIV-2 Vpx^-^). Unless otherwise specified, non-infectious SIV_MAC _Vpx containing VLPs were produced from complete packaging vectors and VSVg pseudotyped (Vpx-VLPs in the text), as we had shown that Vpx is the only protein of SIV_MAC _required for their positive effect [20]. In a typical pre-incubation assay, Vpx-VLPs are added to target cells 2 hrs prior to infection at MOI equivalents comprised between 1 and 2. HIV-1 Vpr and Vpx proteins were expressed from pHA-Vpr and pTG651, respectively [10,20]. When indicated, Vpx proteins were Flagged at their N-terminus.

### Antibodies

Monoclonal antibodies were from the AIDS reagent and reference program of the NIH (anti-SIV CA # 3537), and Sigma (anti-Flag epitope, clone M2).

### Analysis of reverse transcription intermediates

Infections were generally carried out at MOIs comprised between 2 and 10 and PCR analysis carried out on serial five-fold dilutions of cellular lysate, as previously described [33]. Primer sequences were as follows (from 5' to 3', nt within brackets refers to the complete SIV_MAC251_, HIV-1, FIV or MLV sequences; acc. n°D01065, M38432, NC_001482 and Z1118, respectively): minus-strand strong-stop, MSSS, PE103-AGTCGCTCTGCGGAGAGGCTG (nt 507–527) and PE83-TGCTAGGGATTTTCCTGC (nt 789–807) for SIV_MAC251_, AC35-GCCTCAATAAAGC TTGCCTTG (nt 522–542) and AC117-GCATG CTGCTAGAGATTTTCCACAC (nt 616–635) for HIV-1, AC373-GAGTCTCTTTGTTGAG GACTTTTG (nt 217–240) and AC374-TGCG AAGTTCTCGGCCCGGATTCCG (nt 331–355) for FIV, AC311-GTCCTCCGATAGACTGAGT C and AC312-GTAGTCAATCACTCTGAG for MLV; full length, FL, 39-CCGTCGTGGTTGG TTCCTGCCG (nt 878–899) and 40-GCTAGA TACCCAGAAGAGTTGGAAG (nt 294–309) for SIV_MAC251_, AC37-CACTCCCAACGAAGAC AAG (nt 9100–9120) and AC38-CAGCAAGCC GAGTCCTGCGT for HIV-1 (nt 699–708), AC375-TGGGATGAGTATTGGAACCCTGAA G (nt 1–25) and AC376-TTTCTATTGCTCTAG CTTCACTTCC (nt 394–419) for FIV, AC310-CTCAGCAGTTTCTTAAGACCC (nt 8094–8114) and AC267-GATCTGAGCCTATTGATC GATC (nt 44–65) for MLV; 2LTRs circles, PE107-AGCTGCCATTTTAGAAGTAAGCC (nt 664–686) and PE151-TCTGACAGGCCTGA CTTGC (nt 318–336) for SIV_MAC251_, AC34-TCC CAGGCTCAGATCTGGTCTAAC (nt 465–489) and AC35 for HIV-1, AC377-TGTCGAGTAT CTGTGTAATCTTTTTTACC (nt 292–320) and AC378-AAAAGTCCTCAACAAAGAGACTC (nt 217–239) for FIV, AC292-GCTGTTGCAT CCGACTCGTG (nt 60–79) and AC293-CACC GCAGATATCCTGTTTG (nt 7975–7994) for MLV ; mitochondrial DNA, 98-GAATGTCTG CACAGCCACTTTCCAC and 99-GATCGTGG TGATTTAGAGGGTGAAC; actinup-CGAGA AGATGACCCAGATC, actindown-TGCCGCC AGACAGCACTGTG. Probe sequences were: primer PE107 for SIV_MAC251 _MSSS and FL; primer 40 for SIV_MAC251 _2LTRs; primer AC36-TAGAGATCCCTCAGACCCTT (nt 589–608) for HIV-1 MSSS, FL and 2LTRs; primer AC292 for MLV MSSS and FL, AC 312 for MLV 2LTRs; mitoprobe100-TGGGGTTTGGC AGAGATGT; actinprobe-GGAGAAGAGCTA CGAGCTGC.

## Competing interests

The author(s) declare that they have no competing interests.

## Authors' contributions

CG: carried out experiments, data analysis and contributed to writing of the manuscript

LR: carried out initial cloning of Vpx proteins from different SIV strains

LJW: purified blood material, tested proteasome inhibitors cytotoxicity and contributed to virion preparations

JB: provided blood material

DR: provided blood material

JLD: data analysis and study design

AC: study design, data interpretation and supervision
